# Does two dimensional templating allow for the use of reduced-size ancillaries in total hip arthroplasty?

**DOI:** 10.1007/s00264-024-06276-4

**Published:** 2024-08-22

**Authors:** Gregoire Heliere, Guillaume David, Sarah Cypel, Vincent Steiger, Florian Ducellier, Louis Rony

**Affiliations:** Department of Orthopaedic Surgery, CHU d’Angers, 4, rue Larrey, Angers Cedex 9, 49933 France

**Keywords:** Total hip arthroplasty, Preoperative templating, Hip surgery, Single-use ancillary

## Abstract

**Purpose:**

Rising costs in healthcare for total hip arthroplasty (THA) mean that new solutions must be considered, such as the use of single-use ancillaries (SUA). The goal of this study was to assess the accuracy of 2D templating in primary THA for the use of reduced-size SUA. Our hypothesis was that the accuracy of 2D templating in primary THA would be higher than 95%, give or take two sizes.

**Method:**

This single-centre prospective study included all primary THAs performed over two years. Templating was carried out using 2D templating on anteroposterior pelvic X-rays. The template sizes were compared to the implant sizes. The primary endpoint was the rate of coincidence between digitally templated estimates and the actual implant sizes. The secondary endpoint was the difference of accuracy based on patient parameters.

**Results:**

We analysed 512 cases of THA. Accuracy within two sizes was 96.9% for acetabular implants and 98.5% for femoral implants. Accuracy was below the 95% threshold only in patients under 55 and over 85 years old. A BMI above 30.0 kg/m^2^ significantly reduced accuracy but did not fall below the 95% threshold. The operated hip, the type of implant, and the operative indication did not significantly influence templating accuracy.

**Conclusion:**

Using reduced-size SUA with five rasps and five reamers depending on template sizes means that THA can be performed in more than 95% of cases allowing the use of compact single use ancillaries.

**Supplementary Information:**

The online version contains supplementary material available at 10.1007/s00264-024-06276-4.

## Introduction

The rise in the number of THAs (total hip arthroplasties) due to population ageing is a public health issue. In the United States, that number is set to reach 935 000 by 2030 [1]. Solutions must be found to keep healthcare costs under control. Some industrial companies have suggested using single-use ancillaries (SUA) [2]. SUA mean that the ancillary size can be reduced by using only the necessary tools (femoral rasps and motorised reamers), depending on the planned implant size [3]. These ancillaries, smaller in size, mean lower manufacturing and maintenance costs. The solution also helps to reduce logistics costs (including sterilisation costs) for healthcare institutions [4]. To choose the most suitable SUA, it is necessary to know the size of the implants that will be implanted. Preoperative planning is essential. Described by Eggli and Müller [5], preoperative planning helps anticipate intra-operative difficulties, restore hip anatomy (offset, limb length, centre of rotation of the hip) and improve the survival rate of implants [6–8]. Currently, preoperative planning is carried out with a software using digital anteroposterior pelvic X-rays [9]. Scaling the X-rays and ensuring high-quality X-ray images are both key to effective planning [10, 11]. The most common method is using a standard-size radiopaque marker (such as a metal ball placed in the X-ray field near the center of the hip) that will be recognised by the templating software [12, 13]. The goal of this study was to assess the accuracy of preoperative 2D templating for the use of SUA in THA. Our hypothesis was that the accuracy of preoperative planning in the form of 2D templating would be higher than 95%, give or take two sizes.

## Materials and methods

### Population

This study is subject to regulations relating to Research Not Involving Human Subjects (RNIHS). It was approved by a local ethics committee and has been declared to the French Data Protection Authority (CNIL) under number 2022-035.

**Fig. 1:**
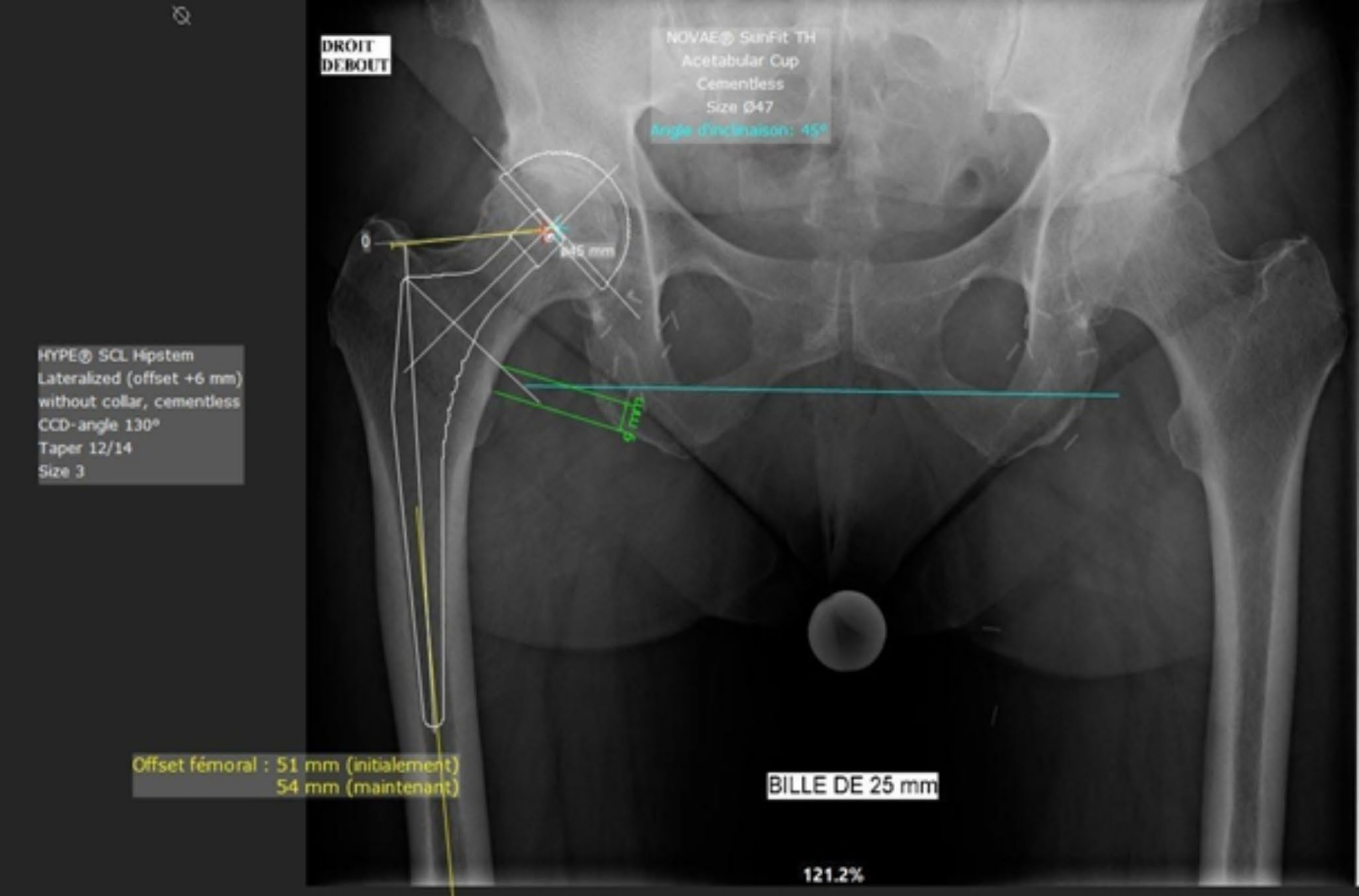
Example of 2D templating

**Fig. 2:**
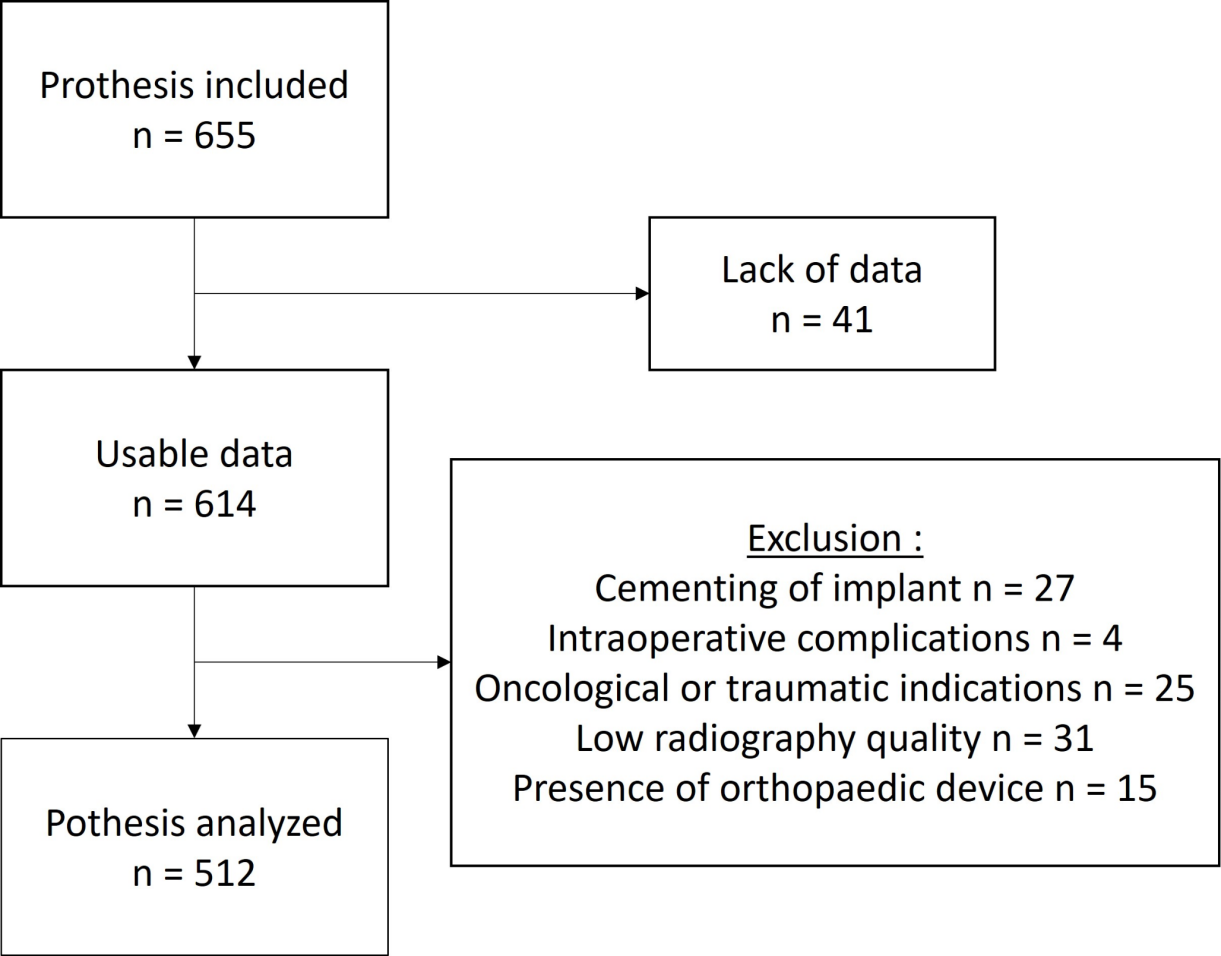
Flowchart

This single-centre prospective study included all primary THAs performed at our hospital between March 2020 and June 2022. The templating was performed by the senior surgeon (SS) responsible for the surgery, using MediCAD 2D Classic Hospital^®^ software (version 6, MediCAD Hectec GmbH, Altdorf/Landshut, Germany) before the surgery (see Fig. 1). The exclusion criteria were: cementing of implant [14], the occurrence of an intra-operative complication other than calcar’s fracture, an indication for surgery in oncology or traumatology, poor-quality radiographs, and the presence of orthopaedic device in the operated hip making it impossible to carry out templating or use of a different implant than those mentioned further down. The flowchart is shown in Fig. 2. The following patient data that could affect templating accuracy was collected: age, sex, BMI, operative indication, operated hip, and type of implant used.

### Implants

The implants used for femoral implants were Hype^®^ cementless stem (SERF, Décines-Charpieu, France) and Optimys^®^ cementless stem (Mathys, Gerzat, France), while for acetabular implants they were Sunfit Novae^®^ cementless dual mobility (DM) acetabular cup (SERF, Décines-Charpieu, France) and Pressfit^®^ cementless RM acetabular cup (Mathys, Gerzat, France).

### Objectives and endpoints

The primary objective was to assess the accuracy of 2D templating in primary THA. The primary endpoint was the rate of coincidence between the actual implant sizes and the digitally templated estimates. We assumed that a 95% concordance within two sizes was possible.

The secondary objective was to identify factors negatively affecting the accuracy of 2D templating in THA and making it difficult to use a SUA tailored to the patient. We analysed the accuracy of 2D templating based on the following parameters: age, sex, BMI, operative indication, operated side, and differences in implant type. We created three groups: Group 1 (exact concordance of sizes, N), Group 2 (concordance give or take 1 size, N+/-1) and Group 3 (concordance give or take 2 sizes, N+/-2).

### Data analyses

Statistical analysis was performed using Systat software (Systa, San José, CA, Version No. 13.00.05). Student’s *T-test* was used between the three groups for quantitative data. The *chi-square* test and Fischer’s exact test were used for qualitative data. Differences were considered significant for a *p*-value below 0.05.

## Results

A total of 655 cases of ATH were included. After exclusion, 512 prosthesis were analysed. The mean age for patients was 70.4 years old ± 12.2 [range, 20 to 93]: with 267 women (52.1%) and 245 men (47.9%). The surgery was performed on the right hip in 259 cases (50.6%) and the left hip in 253 cases (49.4%). In 19 cases (3.9%), the surgery was performed on both hips during the same procedure; in such cases, each side was counted as one prosthesis for the purposes of the study. The mean BMI was 28.0 kg/m^2^ ± 5.4 [range 16.9 to 51.9]. The indication was primary coxarthrosis in 456 cases (89.1%), aseptic osteonecrosis (AON) of the femoral head in 40 cases (7.8%), acetabular dysplasia in ten cases (2.0%), and a different indication in six cases (1.2%). The characteristics of the study population are summarised in Table 1.

**Table 1:** Population characteristics

	*N* (%)
**Sex**	
Male Female	245 (47.9) 267 (52.1)
**Age**	
< 55 years 55–74.9 75–84.9 ≥ 85	51 (10.0) 271 (52.9) 145 (28.3) 45 (8.8)
**BMI**	
< 25.0 kg/m^2^ 25,0–29.9 kg/m^2^ 30,0–34.9 kg/m^2^ ≥ 35.0 kg/m^2^	160 (31.3) 206 (40.2) 99 (19.3) 47 (9.2)
**Hip operated**	
Right Left	259 (50.6) 253 (49.4)
**Indication**	
Primary coxarthrosis Aseptic osteonecrosis Hip dysplasia Others	456 (89.1) 40 (7.8) 10 (2.0) 6 (1.2)
**Implants**	
Hype^®^ stem Optimys^®^ stem DM Novae Sunfit^®^ cup RM Pressfit^®^ cup	351 (68.6) 161 (31.4) 460 (89.8) 52 (10.2)

For femoral implants, accuracy was 42.4% for the exact size (N), 85.4% give or take one size (N+/-1) and 98.5% give or take two sizes (N+/-2). For acetabular implants, accuracy was 40.0% for the exact size (N), 84.6% give or take one size (N+/-1) and 96.9% give or take two sizes (N+/-2).

A significant difference was found for stem templating as it was more accurate in women at N+/-1: 89.1 versus 81.2%; *p* = 0.016. As regards stem templating depending on age, at N+/-1, templating accuracy was worse in patients under 55 years old; *p* = 0.040; and at N+/-2 in patients over 85 years old; *p* = 0.018. BMI was a factor for poorer accuracy for stems when it was higher than 30.0 kg/m^2^; *p* = 0.022; and for acetabular cups for patients with a BMI between 30.0 kg/m^2^ and 34.9 kg/m^2^; *p* = 0.045.

The rest of the analysis did not show any differences in templating accuracy for the following parameters: types of implants used, operated side, and operative indication. Results are summarized in Table 2.

**Table 2:** Results

* = Significant Results Bold = *N*+/-2 results < 95.0%	Stem (%)		cup (%)	
	*N*+/-1	*N*+/-2	*N*+/-1	*N*+/-2
**Sex**				
Male Female	81.2 89.1 *p* = 0.016*	98.0 99.3 *p* = 0.267	84.1 85.0 *p* = 0.864	96.3 97.4 *p* = 0.613
**Age**				
< 55 years 55–74.9 75–84.9 ≥ 85	72.5 87.8 87.6 77.8 *p* = 0.014*	98.0 98.9 100 **93.3** *p* = 0.014*	88.2 83.8 86.2 80.0 *p* = 0.642	**94.1** 98.2 96.6 **93.3** *p* = 0.113
**BMI**				
< 25.0 kg/m^2^ 25,0–29.9 kg/m^2^ 30,0–34.9 kg/m^2^ ≥ 35.0 kg/m^2^	86.3 88.3 80.8 78.7 *p* = 0.182	100 99.0 97.0 95.7 *p* = 0.022*	86.3 84.5 84.8 78.7 *p* = 0.663	98.1 97.6 **93.9** 95.7 *p* = 0.045*
**Hip operated**				
Right Left	87.3 83.4 *p* = 0.267	98.5 98.8 *p* = 1	83.4 85.8 *p* = 0.535	97.3 96.4 *p* = 0.620
**Indication**				
Primary coxarthrosis Aseptic osteonecrosis Hip dysplasia	86.2 80.0 70.0 *p* = 0.157	98.5 100 100 *p* = 1	84.6 87.5 70.0 *p* = 0.801	97.1 95.0 **90.0** *p* = 0.143
**Implants**				
Hype^®^ stem Optimys^®^ stem	87.2 81.4 *p* = 0.959	98.6 98.8 *p* = 0.111		
Novae Sunfit^®^ cup RM Mathys^®^ cup			83.9 90.4 *p* = 0.004*	96.7 98.1 *p* = 0.307

Accuracy at N+/-2 fell below the 95% threshold for stems, in patients over 85 years old and, for acetabular cups, only in patients under 55 and over 85 years old, as well as in patients with a BMI between 30.0 kg/m^2^ and 34.9 kg/m^2^ and patients with acetabular dysplasia.

## Discussion

Our study shows that it is possible to obtain an accuracy over 95% with a margin of error of up to two sizes for femoral and acetabular implants. Our main hypothesis was therefore confirmed.

Using SUA requires accurate templating to be able to choose the ancillary with the right implant size. Digital 2D templating is known to be reliable and stable, according to the literature [15–20] (Table 3). In our study, accuracy within two sizes was over 95%.

**Table 3:** Previous accuracy measurements reported from different studies

Studies Bold = *N*+/-2 results > 95.0%	Stem (%)		Cup (%)	
	*N*+/-1	*N*+/-2	*N*+/-1	*N*+/-2
**Shichman** et al. (2020)	61.4	81.2	77.2	93
**Thirion** et al. (2020)	90	**100**	93	**100**
**Schmidutz** et al. (2012)	88.7	**98.8**	75.8	93.3
**Holzer** et al. (2018)	87	**97.6**	78	94.7
**Si** et al. (2015)	84.4	93.3	78.9	**95.6**
**Shaarani** et al. (2013)	75	**98**	80	**98**
**Kumar** et al. (2009)	78	91	91	**100**
**Mirghaderi** et al. (2022)	61	78.6	63.9	83.1

2D templating is still the gold standard. It is inexpensive and easily accessible, and it induces a low radiation dose for patients [21]. The main limitations are the need to place the standard radiopaque marker in the correct position on the X-ray for the purposes of calibration [10, 20] and the lack of 3D assessment of hip anatomy [22, 23]. 3D templating is therefore more accurate [24–26] notably when used with artificial intelligence 3D-planning [27]. It requires performing a hip CT scan or using EOS^®^ biplanar X-ray imaging [28]. EOS^®^ is not available at all healthcare institutions [29] and CT scans involve more radiation, are more expensive [30] and are not performed systematically for coxarthrosis. However, would the higher accuracy of 3D templating for the use of reduced-size ancillaries not compensate for the higher treatment costs and the higher radiation dose? A medical and economic study of the overall cost would need to be performed to answer that question.

SUA are widely used for scan-based osteotomies, trauma surgeries and knee arthroplasties. But barely used in hip arthroplasties. This could be explained by the need of a more durable and resistant material to make rasps and reamers than cutting guide. Healthcare institutions might still find SAU useful because it lowers sterilisation costs [3] and simplify logistics for operating teams [31]. For industrial companies it’s reducing their logistics and maintenance costs for ancillaries. Some studies also mention the benefits regarding septic risk and operating time [32]. The main barriers to using SUA are the higher purchase price and the fact that using them creates more waste [33]. Currently, no prosthesis manufacturer makes recyclable or reusable SUA. Innovation in this direction would have both environmental and financial benefits and could help SUA become more popular.

2D templating has some limitations. In our study population, we examined the factors that affect the reliability of 2D templating. The surgeon’s level of experience is a major factor. Carter et al [34]. found a higher accuracy in the case of senior surgeons compared to junior surgeons (JS) for cementless primary THAs. Other studies have shown similar findings, for example the studies by Shichman et al [19]. and Montiel et al [35]. Thirion et al [36]. showed that an engineer who is used to templating is able to produce results comparable to those of a senior surgeon. Mastering the templating software more so than the surgical procedure is a major factor. This may partly explain our promising findings given that the templating was carried out by experienced surgeons.

Our findings showed that accuracy for femoral and acetabular implants at N+/-2 fall below the 95% threshold for patients in the extreme age groups (under 55 or over 85 years old), with a significant difference for stems in patients over 85 years old. The difference in bone cortical density could be a factor that misled surgeons, who tended to overestimate the implant size in younger patients and underestimate it in older patients. The significant difference in accuracy for stems between the sexes was related as much to the difference in bone density between the sexes as to the anatomy of the proximal femur [37]. These differences are better assessed using 3D templating [38].

In our study, accuracy was still beyond the 95% threshold regardless of the BMI, with a significant difference for stems between patients who had a BMI of less and more than 30.0 kg/m2. Patients with a BMI under 29.9 kg/m^2^ had a 99.5% accuracy for the stem compared to 96.6% with a BMI over 30 Kg/m^2^. Sershon et al [39]. found mean accuracy, give or take one size, to be 97.1% for stems and 99.1% for acetabular cups, without showing a difference between the different categories of BMI. Ramme et al [11]. confirmed the difficulties relating to correctly placing the calibration marker in patients with obesity. Soft tissue moves the metal ball away from the centre of the joint and increase peripheral distortion in the X-ray [10] which causes scaling errors and therefore errors in the size of the implants. The patient profile with the highest accuracy is therefore women aged between 55 and 85 years old with a BMI under 30 kg/m^2^. In our study, this applied to 167 individuals, or 32.6% of the overall population or 63.7% of women.

Differences in implant design could be a source of inaccuracy in templating. We did not find any differences based on the different types of implants used (short stem/quadrangular stem and DM/single mobility acetabular cup). The lack of difference had already been confirmed by Schmidutz et al [40]. and Mirghaderi et al [41]. This finding suggests the SUA could be used with all implants on the market.

The strengths of our study include the high number of patients included and the reflection of our day-to-day practices given that we included all the THAs performed in our department during the relevant period. Only the study by Holzer et al. [16], with 632 patients, had a larger population than ours and showed similar findings. The study by Mirghaderi et al. [41], with 391 patients, showed poorer findings than ours, but the population had a much lower mean BMI and their surgeons were less experienced in performing templating.

The limitation of our study lies in the profile of our study population which, due to our exclusion criteria, mainly involved cases of primary coxarthrosis and AON. We chose to exclude perioperative complications resulting in cementation of the femoral stem or the use of other implants than templated. This could be a bias but we only had four out of 614 patients excluded for this reason. We kept in the study the perioperative calcar’s fractures only needing cerclage wires. We noted only three of those fractures in our population. It may be caused by an error in templating with an overestimation of the size of the femoral stem [38]. The incidence was too low to measure its impact. Our study included only a few cases of hip dysplasia. These are the ones for which it is most difficult to carry out templating [42]. SUA do not yet seem a viable option when it comes to such indications. The use of 3D templating is a solution to consider for such indications because it ensures a higher level of accuracy.

## Conclusion

Our study showed that 2D templating on digital X-rays ensures an accuracy above 95%, give or take two sizes, which allows for the use of reduced-size SUA (5 rasps, 5 reamers and 3 necks). We concluded that SU ancillaries are most suitable for female patients with a BMI under 29.9 kg/m2 and aged between 55 and 85 years old. Other studies on the overall cost of templating and its 3D alternatives must be carried out to address the issue of the rising number of THAs in the years to come.

## Electronic supplementary material

Below is the link to the electronic supplementary material.

Supplementary Material 1
